# Idiosyncratic Drug-Induced Neutropenia and Agranulocytosis in Elderly Patients

**DOI:** 10.3390/jcm9061808

**Published:** 2020-06-10

**Authors:** Noel Lorenzo-Villalba, Maria Belen Alonso-Ortiz, Yasmine Maouche, Abrar-Ahmad Zulfiqar, Emmanuel Andrès

**Affiliations:** 1 Diabètes et Maladies Métaboliques, Service de Médecine Interne, Hôpitaux Universitaires de Strasbourg, 67000 Strasbourg, France; noellorenzo@gmail.com (N.L.-V.); yasmine-hafsa.maouche@chru-strasbourg.fr (Y.M.); abzulfiqar@gmail.com (A.-A.Z.); 2 Servicio de Medicina Interna, Hospital Universitario de Gran Canaria Dr Negrin, 35000 Las Palmas de Gran Canaria, Spain; belalor@yahoo.es

**Keywords:** agranulocytosis, drug-induced agranulocytosis, idiosyncratic agranulocytosis, neutropenia, elderly patients, non-chemotherapy drugs, drug adverse reactions, hematopoietic growth factors

## Abstract

Agranulocytosis is a rare, but serious and life-threatening hematologic disorder in elderly patients. Idiosyncratic drug-induced agranulocytosis (IDIA) has been classically defined by a neutrophil count below 0.5 × 10^9^/L. The annual incidence of IDIA in Europe is about 1.6–9.2 cases per million inhabitants. Increasing age and female sex have been considered as risk factors for the development of this condition. Besides, it is well known that older people take on average more drugs than younger people. This condition is most often associated with the intake of antibacterial agents, antiplatelets, antithyroids, antipsychotics, antiepileptics and nonsteroidal anti-inflammatory drugs (NSAIDs). Initially, agranulocytosis may present without symptoms, but may quickly progress to a severe infection and sepsis. The causative drug should be immediately stopped. In febrile patients, blood cultures and where indicated, site-specific cultures should be obtained and early treatment with empirical broad-spectrum antibiotics started. Even with adequate treatment, the mortality rate is higher in elderly patients reaching up to 20%. Hematopoietic growth factors have proven to be useful as they shorten the duration of neutropenia. However, data on neutropenia and agranulocytosis in the elderly meeting the criteria of evidence-based medicine are still poor in the literature. This review analyzes the results of our experience as well as other published studies of the universal literature.

## 1. Introduction

Between 2015 and 2050, the proportion of people aged 60 and over will almost double in the world, from 12% to 22%. By 2050, the World Health Organization (WHO) expects the world’s population aged 60 and over to reach 2 billion people, up from 900 million in 2015 [1]. According to the WHO, aging is the result from physical and psychological changes in the organism. These changes lead to disturbances related to the reduction of functional capacities, but also to the possible sequelae of the diseases from which the person may have suffered throughout their life. It is important to highlight that each individual ages differently.

Some will remain in good health for a long time, while the others considered as frail may age less well. Thus, appears the notion of frailty, with many scales making it possible to classify the elderly differently. Aging is associated with impaired adaptation and homeostatic mechanisms leading to the increased susceptibility to environmental and internal stress with an increased risk of disease and death. Physiological aging is often associated with chronic pathologies, which require appropriate management. Drug management in the elderly is very complex due to changes in the pharmacodynamics and pharmacokinetics of drugs and the presence of multiple comorbidities. 

Moreover, elderly patients are often exposed to many drugs and therefore to drug interactions. The side effects of the latter are known and expected or unpredictable in this context. One of the problems rarely discussed in literature is idiosyncratic drug-induced agranulocytosis in the elderly.

The majority of cases of neutropenia in adult individuals is acquired and may result from the decreased granulocyte production or its increased destruction. The decrease in the granulocyte count can be predictable and dose related, as in cytotoxic chemotherapy, or the result of an idiosyncratic reaction. Of note, leukopenia and granulocytopenia are used interchangeably with neutropenia in medical practice, even though they are somewhat different. In fact, leukopenia specifically refers to a low white blood count from any cause, while granulocytopenia point to granulocyte reduction. 

Besides, the term agranulocytosis strictly means the absence of granulocytes; however, the term is frequently used to indicate severe degrees of neutropenia. Drug-induced agranulocytosis, or severe neutropenia, has been classically defined by a neutrophil count below 0.5 × 10^9^/L, usually health impairment and often severe infections [2]. Clinical manifestations vary from asymptomatic cases to those presenting fever, chills, a sore throat and a variety of severe infections (pneumonia, septicemia or septic shock). Indeed, several factors have been associated with a poor prognosis: age ≥ 65 years, the presence of comorbid conditions such as chronic renal failure or systemic inflammatory diseases, absolute neutrophil count at diagnosis below 0.1 × 10^9^ cells/L and severe infections [3,4,5].

Currently, the incidence of non-chemotherapy drug-induced agranulocytosis, also called idiosyncratic drug-induced agranulocytosis, is not accurately known, but it is considered a rare and serious condition. Increasing age and female sex have both been considered risk factors for the development of this condition. That is why it is crucial to give special attention to elderly individuals.

## 2. Definition

Agranulocytosis was initially defined as an important decrease in the number of absent granulocytes in circulating blood, resulting in at least an absolute neutrophil count of <0.5 × 10^9^ cells/L or less than 0.1 × 10^9^ cells/L in most patients [6,7]. Drugs have been related to the development of agranulocytosis but they do not explain it in all cases. Agranulocytosis could be the result of non-chemotherapy drugs, immune modulator agents or biotherapies. The latter results in “idiosyncratic” drug-induced agranulocytosis. The causative agent could be the drug itself or one of its metabolites. Idiosyncratic drug-induced agranulocytosis, or severe neutropenia, has been classically defined by a neutrophil count below 0.5 × 10^9^/L, usually health impairment, and often severe infections [2].

However, the definition and criteria of drug imputability for idiosyncratic chemical drug-induced neutropenia and agranulocytosis, derived from an international consensus meeting and the International Agranulocytosis and Aplastic Anemia Study (IAAAS) are currently used [8,9,10]. This definition includes: a. “severe” neutropenia as defined by a neutrophil count <0.5 × 10^9^/L; and b. agranulocytosis by a neutrophil count <0.5 × 10^9^/L with the existence of a fever and/or any signs of infection or a neutrophil count less than 0.1 × 10^9^ cells/L.

Regarding the criteria of drug imputability, it is considered: a. the onset of agranulocytosis during treatment or within 7 days after exposure to the drug; b. with a complete recovery in neutrophil count of more than 1.5 × 10^9^/L within one month of discontinuing the drug; c. recurrence of agranulocytosis upon re-exposure to the drug; and d. the exclusion of other conditions such as a history of congenital neutropenia or immune mediated neutropenia, recent infectious disease (particularly recent viral infection or severe Gram-negative infections), recent chemotherapy and/or radiotherapy and the existence of an underlying hematological disease [8].

## 3. Incidence and Risk Factors

Idiosyncratic drug reactions have an estimated incidence of 1/10,000 to 1/100,000, but the actual incidence of idiosyncratic drug-induced agranulocytosis is not well known [5]. Several studies have shown incidence rates from 1.1 up to 15.4 cases per million in the population per year [11,12]. The annual incidence in Europe ranges from 1.6 to 9.2 cases per million in contrast to the USA, where reported rates range from 2.4 to 15.4 per million per year [9,13,14,15]. In an observational study carried out in France between 1996 and 2003, the annual incidence of symptomatic cases was approximately 6 per million [15]. Another recent French study from 2015 to 2017 described 41 patients with suspected non-chemotherapy drug-induced neutropenia without any clear sex predominance [16].

In the IAAAS study in Europe and Israel from 1980 to 1986, the incidence increased with age (10% occurred in children and young adults and >50% in patients over age 50) [9,10]. In a study conducted in our University Hospital (*n* = 201 patients), 67% of patients were aged 65 years and over [15]. The incidence of this entity rising with age probably reflects the increased use of medications in this population. According to some reports, a high proportion of elderly patients take four to five medications on a regular basis [15]. Thus, polypharmacy is a challenge when determining the causative or likely causative drug [16]. Another important risk factor in elderly individuals is the high incidence of chronic renal failure, and in consequence, drug excretion impairment in the urine or the concomitant use of certain medications such as probenecid in patients taking captopril which have also been related to agranulocytosis [17,18]. However, data on neutropenia and agranulocytosis in the elderly meeting the criteria of evidence-based medicine is poor in the literature.

Besides, in the previously mentioned study [15,16], agranulocytosis was almost one to two times more frequent in women and this sex difference may be probably due to the greater consumption of drugs at higher risk of causing neutropenia by adult females. This explanation is based on the fact that there was no female preponderance in children [19].

Other risk factors such as human leukocyte antigen (HLA) or the presence of autoimmune diseases have been described. The HLA-B38 phenotype and the combined alleles DR4 and DQw3 have been related to an increased frequency of clozapine-induced agranulocytosis [20]. Conversely, the occurrence of HLA-B35 may have a protective role in certain ethnic groups from developing clozapine-induced agranulocytosis [21]. The HLA DRB1* allele appeared to be strongly associated with susceptibility to methimazole-induced agranulocytosis in a Japanese patient with Graves’s disease [22]. Besides, the presence of HLA-B27 has been related to the development of agranulocytosis during treatment with levamisole [23].

## 4. Drugs Involved

Many drugs have been described as potential causes of severe neutropenia or agranulocytosis based on case reports, spontaneous reports, registries, cohort studies, and population and case control studies. Based on the available evidence, drugs can be classified as definite, probable, or possible causes of neutropenia. (Table 1).

The risk of agranulocytosis is low for most of these but this risk is especially high with clozapine, thionamides (antithyroid drugs), sulfasalazine, cotrimoxazole, ticlopidine, gold salts, phenylbutazone and penicillamine [24,25,26]. In the case of antithyroid drugs, a risk of one per three users has been reported [27]. Clozapine has been described to produce agranulocytosis in about 1% of patients, especially in elderly individuals and females during the first three months of treatment [15].

In this population, the effects of aging modify the pharmacokinetic and pharmacodynamic parameters. The effect of age on drug sensitivity varies from drug to drug; it depends on the variation in the number of receptors and/or the variation of the effect at the level of these ones and/or the variation post-receptor effect. 

Other drugs such as angiotensin converting enzyme (ACE) inhibitors, amodiaquine, histamine H2-receptor antagonists, nonsteroidal anti-inflammatory drugs, tocainide, procainamide, flecainide, aminoglutethimide, deferiprone, and dapsone have been incriminated [18,28,29,30,31,32,33,34,35]. In a previous study conducted in our hospital, the most frequently causative drugs were antibacterial (β-lactam and cotrimoxazole), ticlopidine, and antithyroid drugs [27]. In the Berlin Case–Control Surveillance Study (FAKOS) aimed to identify pharmaceuticals with an increased risk for this condition, the highest odds ratios were observed for clozapine, sulfasalazine, and thiamazole [25]. A recent study published by Spanish group showed metamizole and piperacillin/tazobactam followed by dexketoprofene, linezolid, amoxicillin/clavulanic, and meropenem as the most common causative drugs [36].

All biotherapies on the market, in the USA and Europe, have also been associated with neutropenia and this has been especially well documented for TNF-α inhibitors, tocilizumab, rituximab and alemtuzumab [37]. For most of these drugs, the overall neutropenia risk is estimated to be around 10%, but it appears minimal (<1%) for severe neutropenia and agranulocytosis [27].

## 5. Pathophysiology 

Two mechanisms explaining drug-induced neutropenia and/or agranulocytosis include: drug-dependent immune-mediated destruction of circulating neutrophils, drug-induced antibodies and direct toxic effects upon marrow granulocytic precursors [6,9].

The immune-mediated drug-induced agranulocytosis hypothesis considers that the drug, or more likely a reactive metabolite of the drug, irreversibly binds to the neutrophil membrane. The reactive metabolite in some cases may result in the production of antibodies or T cells directed against the altered membrane structure; in others, true anti-neutrophil autoantibodies are produced that do not require the presence of the drug [38]. Propylthiouracil, amodiaquine and flecainide are some of the drugs known to act as haptens in the production of autoantibodies [5,31,39,40]. Of note, dipyrone may also induce the production of anti-neutrophil antibodies [41]. The presence of these antibodies can be demonstrated using the ELISA test, but it requires the presence of the suspect drug/metabolites [42]. The failure to reproduce such a mechanism experimentally could be due to either antibody specificity for a metabolite of the drug or a low antibody titer [43].

Direct and indirect toxicity have been the mechanisms evoked in other drug groups [44]. The complex metabolic pathways that detoxify drugs and chemicals are genetically regulated and genetic polymorphism could be at the origin of different levels of expression of genes encoding enzymes that generate or destroy toxic intermediate compounds. Direct toxicity is the mechanism described with phenothiazines, especially chlorpromazine and procainamide, dapsone, and clozapine [45,46,47,48].

In other reported cases, intermittent exposure is implicated. This suggests an immune-mediated mechanism involving cytotoxic T cells, oxidative modifications of the drug, and haptens [6,49,50].

The onset is often delayed and can even appear 30 days after the drug has been discontinued. The delay is much shorter if the causative drug is a re-challenge [5].

## 6. Clinical Manifestations

The majority of cases of idiosyncratic drug-induced neutropenia and agranulocytosis present within the three to six months after beginning the causative drug. The clinical presentation depends in part upon the etiology and pathogenesis of the drug-induced agranulocytosis. The onset could be abrupt with significant symptoms days to weeks after starting the drug in the immune-mediated mechanism. In contrast, neutropenia presents insidiously and asymptomatic when direct or indirect toxicity is the leading mechanism [51,52,53].

The first case reports described edema, necrosis and the destruction of the pharynx preceding prostration and death [2]. Patients usually present with oral ulcerations with or without fever, sore throat, stomatitis, diarrhea and general malaise [6,13]. Arthralgias and myalgias are also described. In the elderly, the initial clinical presentation may be mildly symptomatic, masked, and may progress quietly to a severe infection. As in patients receiving chemotherapy, the occurrence of infections depends on the degree and duration of the neutropenia and the patient phenotype (age, medical history and type of comorbidities). This is of relevant importance in elderly patients who usually present several comorbid conditions. Aging is also characterized by a loss of functional units over time and by a disruption of certain regulatory processes; all of which result in a reduction in the body’s ability to adapt to physiological stress. The pharmacokinetic and pharmacodynamic parameters are thus modified by the effects of aging.

In our drug-induced agranulocytosis cohort study, conducted in 61 elderly subjects over 75 years of age, only two patients (3.4%) remained asymptomatic, while a severe clinical course with either septicemia or septic shock was seen in 1:4 of them. The sex ratio was 2:1 and the mean neutrophil count at diagnoses, 0.15 + 0.17 × 10^9^/L. The main clinical features were isolated fever, septicemia and septic shock and documented infection (pneumonia and other deep infections) in this order. (Table 2) [54]. The asymptomatic patients were those with the shortest neutropenia duration. Over 25% of our patients presented features of severe sepsis, septic shock and/or systemic inflammatory response syndrome (SIRS) and 17.5% required intensive care. However, in The Berlin Case–Control Surveillance Study (FAKOS), which included 51 Berlin hospitals between the years 2000 and 2010, only 12.5% of cases progressed to sepsis and more of 60% had a non-systemic infection. However, it must be indicated that 70% of cases were under 70 years [25].

In elderly patients, a low granulocyte count (<0.5 × 10^9^/L) could be accompanied by other hematological abnormalities, such as anemia (20–30%) and thrombocytopenia (10%) [8,15]. These findings justify routine bone marrow examination in elderly individuals, in order to rule out other underlying conditions and predict the duration of the neutropenia [11]. The bone marrow typically shows a normal or mildly reduced cellularity, contrasting with the absence of myeloid precursor cells. The so-called myeloid blocking refers to the lack of any mature myeloid cells whereas immature forms to the myelocyte stage remain preserved. This aspect may be the result of either a drug/antibody effect, specifically on mature cells, or represent an initial stage of recovery [55,56]. In the case of a lack of myeloid precursors, granulocyte recovery will be unlikely before 14 days. This is of significant importance when treating elderly people as the risk of severe infection may exponentially rise. One of the first signs of hematologic recovery is the presence of a blood monocytosis. In practice, a trained cytologist is needed to differentiate on the bone marrow between drug-induced agranulocytosis and certain myelodysplastic or early leukemic syndromes.

## 7. Differential Diagnosis

Differential diagnosis is often not easy in elderly patients, as in this populations many conditions may induce absolute neutropenia. However, physicians should primarily rule out: a. nutritional deficiencies (cobalamin, folic acid and copper); b. qualitative and quantitative major nutritional deficiencies (e.g., marasm and Kwashiorkor); c. bone marrow failure initially presenting with neutropenia; d. neutropenia secondary to hypersplenism, and e. neutropenia secondary to several infections, especially viral infections for moderate neutropenia (e.g., Citomegalovirus, Parvovirus B19) and severe bacterial infections for severe neutropenia (e.g., *Salmonella* sp., Gram-negative bacteria) [6,57,58]. Secondly, it could be necessary to perform a bone marrow aspiration to rule out myelodysplasia, leukemia or medular invasion (e.g., stomach cancer, myeloma).

Among patients presenting with infection, it may not be easy to determine whether the infection is the consequence or the cause of neutropenia. However, this is of particular importance if the medication is crucial to the patient’s management. Bone marrow examination can be of help in these cases; if the cellularity is normal with a late myeloid arrest, the danger from neutropenia is likely significantly reduced, and re-challenge or even continuation of the drug with careful monitoring may be possible. Other less frequent differential diagnoses include Felty’s syndrome and systemic lupus erythematosus: In the former, neutropenia results from the peripheral destruction of polymorphonuclear cells and for the latter it is often drug associated [59].

## 8. Prognosis and Mortality

Three decades ago, the death rate from idiosyncrasy drug-induced agranulocytosis was between 10 and 30% in European studies, but this rate has been progressively decreasing, being to date <5% in our cohort study, probably due to improvements in the management and treatment of this disorder [14]. Mortality rates have dropped to <10%, compared to the 10–16% until the 1990s in some European studies [15]. In elderly patients, a death rate closer to 20% was currently reported by several authors (with regard to a sub-population study) [4,15].

Several prognosis factors have been described. They are of special importance in elderly patients as they include: (a). age >65 years; (b). pre-existing comorbidities (especially defined kidney failure serum creatinine > 120 µmol/L); (c). absolute neutrophil count at the time of diagnosis < 0.1 × 10^9^/L; and (d). the occurrence of severe infection (sepsis and septic shock) [3,4,5,14]. In a Spanish study, renal insufficiency at diagnosis and the development of bacteremia were associated with a poor prognosis as evidence of severe infections, in association with a neutrophil count < 0.1 × 10^9^/L [60]. Moreover, advanced age, decreased leucocyte count, lymphocytopenia, bone marrow myeloid hypoplasia, increased percentage of bone marrow plasma cells and shock were all associated with a poor prognosis only in the univariate analysis. An independent analysis of the myeloid cellularity at diagnosis showed an inverse correlation with the time to recovery of the granulocyte counts (*r* = −0.43; *p* = 0.001). In our experience, the highest mortality rate is observed in older patients, as well as in those with renal failure, bacteremia and shock at diagnosis. In addition, neutrophil count of <0.1 × 10^9^/L at diagnosis, septicemia and/or septic shock were variables significantly associated with a longer time to neutrophil recovery. In contrast, the use of hematopoietic growth factors is associated with a shorter time to neutrophil recovery [13,54].

## 9. Management

Management could be difficult in elderly patients where multiple comorbid conditions may coexist in the setting of frailty. Frailty is a major problem during aging and it is multifactorial. It is also considered a risk factor for the occurrence of adverse events, dependence and morbi-mortality in the elderly. Polypharmacy is one the elements closely related to frailty. Despite this, no specific protocols of management of idiosyncratic drug-induced neutropenia and agranulocytosis in the elderly have been established. Caution should be considered in elderly patients with renal failure, where doses should be adjusted to their renal function. This is also to consider in patients with a previous history of heart failure, malnourished or with proven hypoalbuminemia when choosing adequate doses of drugs.

The causative drug should be withdrawn even in asymptomatic patients once agranulocytosis was found [4,61]. Neutropenia may resolve within one to three weeks after the cessation of the causative drug, but there is important interpatient variability [62,63]. In elderly patients, as the result of self and polymedication, it could be difficult to determine the causative drug [64]. It is necessary to investigate the use of eye drops, cream or pomades and other possible self-medication. The pharmacological history should be chronologically and carefully examined in order to detect the causative agent. The prevention of secondary infections is important, especially in areas such as the mouth, skin and perineum. The causative drug should be withdrawn and the therapeutic class changed, instead of using a same class drug, especially if they share similar chemical structures and/or antigenic determinants.

Bone marrow aspiration and biopsy may be helpful, but probably of less interest in cases of isolated agranulocytosis in patients taking a high-risk drug. If the patient is febrile, cultures of blood, urine, sputum, and other suspected sites of infection should be taken and broad-spectrum intravenous antibiotics immediately started. This is generally the best therapeutic choice but may be adapted based on local antibacterial resistance, site of infection and the patient’s clinical condition [3,63]. However, if an antibiotic is suspected as the offending drug, potential antibody cross reactivity should be considered and antibacterials carefully chosen. The elective treatment are cephalosporines or even cephalosporines with aminoglucosides or cephalosporines with fluoroquinolones [15,64].

Granulocyte colony-stimulating factor (G-CSF) represents another important therapeutic tool, even though their efficacy has not been conclusively proven. An important number of non-randomized studies have reported excellent results with the use of granulocyte colony-stimulating factor (G-CSF) in patients with drug-induced agranulocytosis. Their use has been associated with shorter recovery times and lengths of hospitalization, and less antibiotic use compared with historic or concurrent controls [65,66,67,68,69,70]. One study showed a neutrophil recovery (≥1.0 × 10^9^/L) within one to 15 days (median four days) after the initiation of G-CSF in doses of 4 to 10 µcg/kg per day [66]. Another study evaluated the effects of G-CSF on recovery, at four and 24 h after injection in 37 patients. In 25 patients with mild neutropenia, recovery was noted within four hours and remained normal. Daily G-CSF injections were required from 2 to 11 days in the rest of the patients in this series [67]. In our experience, the use of G-CSF or granulocyte macrophage colony-stimulating factor (GM-CFS), at a mean dose of 300 µcg/day, was also useful in life-threatening idiosyncratic drug-induced agranulocytosis in the elderly [3,6,15]. Of note, no important side effects or toxicity such as increased bone pain and significant leukocytosis were found during treatment with G-CSF. Besides, in the multivariate analysis, the use of G-CSF was an independent variable positively affecting time to hematological recovery. Another study showed similar results to ours, in patients aged ≥65 years, and earlier hematological recovery was observed compared with those who did not receive this treatment [71]. Treatment with G-CSF can be discontinued when the total white blood cell count exceeds 10 × 10^9^/L [3]. In contrast to what was previously mentioned, a prospective randomized study in patients presenting antithyroid-related drug-induced agranulocytosis did not show any further benefit but this probably resulted from the sub-therapeutic dose of G-CSF of 1 to 2 µcg/kg per day [72].

The transfusion of granulocyte concentrates might only be considered in exceptional situations or life-threatening antibacterial-resistant infections such as perineal gangrene or in those inappropriate responding to a well-adapted antibiotic therapy.

Finally, it is necessary to declare this event to the relevant drug agencies. Likewise, the patient and the attending physician must be informed and it would be advisable to indicate it in the medical record and the medication sheet to avoid using these drugs.

## 10. Prevention

There is presently no effective way or established protocols to detect patients at high risk for drug-induced neutropenia or agranulocytosis. White blood cell count and white cell differential monitoring have been used in the early detection of this entity, while prescribing several drug regimens, such as clozapine and thionamides. The value of this monitoring is uncertain and controversial. However, they could be useful in reducing morbidity and mortality in elderly individuals treated with drugs at high risk of drug-induced neutropenia or agranulocytosis.

Aging is a slow, progressive and uneven process depending on the physical, psychic, genetic and environmental variability of each person. While older people experience “successful” or “usual” aging, others will progressively lose autonomy. In this special population, polypharmacy will have to be carefully examined. Although the most commonly used definition of polypharmacy involves five or more drugs, the definitions remain variable. Numerical definitions of polypharmacy do not take into account the specific comorbidities and make it difficult to assess the safety and relevance of treatment in the clinical practice. That is why it will be of help to use “appropriate polypharmacy” as a comprehensive approach to the assessment of drug use in the context of current co-morbidities in order to optimize outcomes in terms of management for the elderly patient. Reducing polypharmacy may have a direct beneficial effect by reducing the prevalence of drug interactions and also idiosyncratic drug-induced agranulocytosis. The STOPP AND START (Screening Tool of Older Persons’ Prescriptions/Screening Tool to Alert to Right Treatment) is considered a useful tool to optimize the prescription of medication in elderly individuals [73].

The advantage of this tool is that, while targeting the most commonly prescribed drugs in geriatrics, it would allow doctors to detect over or insufficient treatment, as well as inappropriate treatment. This will also permit to identify drug interactions and drug-comorbidity interactions leading to side effects [73].

An important element to take into consideration when managing this entity in elderly patients is the so-called frailty syndrome. Described since the 1970s, the frailty syndrome is a dynamic and evolving geriatric concept, involving many dimensions of everyday life and leading to a risk of developing a loss of autonomy. It corresponds to a precarious state of equilibrium linked to the reduction of physiological reserves linked to aging and is responsible for an inability to respond to physical, psychological or social stress. Its management requires medical, social and psychological interventions [74].

The increasing number of dependent elderly people and its health and economic consequences, makes frailty a major public health issue requiring immediate target preventive interventions.

Frailty is not a spontaneously resolving process, but could be reversible if recognized early enough and targeted intervention is conducted [75]. Screening for frailty in primary care combined with the intervention of a geriatric team if needed, and has been proven to limit or even stop the development of a syndrome of frailty [76].

Similarly, a meta-analysis of randomized controlled trials showed a reversibility of this status in frail patients who underwent physical training [77]. However, frailty remains difficult to assess in primary care as the result of the multitude of definitions included and the diagnostic tools currently available. Moreover, not all of these instruments have been validated in general medicine and their implementation is not systematically adapted to it [78].

Frailty occurs when multiple physiological systems decline, resulting in cellular repairment mechanism and homeostasis failure [79]. As the world’s population ages, frailty is moving to the forefront of health and medical research. The early detection of frailty will help minimize its complications and reduce multimorbidity. 

Fried scale is widely known, but it requires measurements difficult to perform in daily medical practice. Besides, there are no psychosocial components in this scale [79].

PRISMA-7 contains seven simple self-reported components. A total score≥ 3 is deemed as frailty [80]. It has a good accuracy in identifying frailty in community-dwelling older people [81], but ittends to over-screen frailty [82].

The Gerontopole Frailty Screening Tool (GFST) comprises two parts: a questionnaire and a subjective clinician’s evaluation of frailty status [83,84]. One limitation of this scale is the lack of specific management once frailty is identified. Moreover, most of the items are subjective. 

The Frailty Index (FI) of Accumulative Deficits (FI-CD) is another well-known frailty screening tool. It has been validated and has a higher predictive ability of adverse clinical events than other frailty scores in both hospital and community settings [85,86], but it is time consuming. There is also a Frailty Index derived from Comprehensive Geriatric Assessment (CGA). It is used as a clinical standard for frailty assessment and has been found to be highly associated with the FI-CD [87].

The border between aging and frailty is narrow but they share similar pathophysiological mechanisms [88,89,90]. The frailty syndrome represents a transitional dynamic process relating to functional decline and aging [90,91].

## 11. Conclusions

Idiosyncratic drug-induced agranulocytosis is rare in elderly patients but it is a life-threatening hematologic disorder. In this population, the progression to severe sepsis is significant and more serious than in younger patients. The management of idiosyncratic drug-induced agranulocytosis or severe neutropenia begins with the immediate withdrawal of any potentially causative drug. In elderly patients, the patient’s medication history must be carefully and chronologically obtained in order to focus on the suspected agent. In this sense, the adequate monitoring of certain pharmacotherapies would be of help. Unfortunately, the pathophysiological mechanisms are complex and still poorly understood. Nonetheless, today there is a better knowledge and management of this disorder. In all cases, an exhaustive pharmacovigilance investigation must be conducted. The declaration of this side effect to the pharmacovigilance authorities must also always be done. This rare and serious disorder requires multidisciplinary care involving a general internal medicine specialist, geriatrics, hematologists, biologists, immunologists and pharmacologists.

## Figures and Tables

**Table 1:** Major medications with a definite association with agranulocytosis.

Therapeutic Class	Drugs
Antibiotics	Macrolides, Sulfonamides, Chloramphenicol, Trimethoprim-Sulfamethoxazole (Cotrimoxazole), Semisynthetic Penicillin, Vancomycin, Cephalosporins, Dapsone
Psychotropic Drugs	Clozapine, Phenothiazines, Tricyclic and Tetracyclic Antidepressants, Meprobamate, Cocaine/Heroine (Adulterated with Levamisole)
Cardiovascular Drugs	Antiarrhythmic Agents (Tocainide, Procainamide, Flecainide), Ticlopidine, Angiotensin converting enzyme inhibitors (Enalapril, Captopril), Propranolol, Dipyridamole, Digoxin, Thiazides, Acetazolamide, Furosemide, Spironolactone, Tolbutamide
Dermatologic Drugs	Dapsone, Isotretinoin
Anticonvulsants	Phenytoin, Carbamazepine, Ethosuximide, Valproate
Anti-Inflammatory Drugs	Sulfasalazine, Nonsteroidal Anti-Inflammatory Drugs, Gold Salts, Penicillamine, Phenylbutazone, Antipyrine, Dipyrone, Phenacetin
Antithyroid Drugs (Thionamides)	Methimazole, Carbimazole, Propylthiouracil
Sulfonylureas	Chlorpropamide; Tolbutamide
Iron chelating Agents	Deferiprone
Gastrointestinal Drugs	Sulfasalazine, Histamine H2-Receptor Antagonists
Antimalarial Drugs	Amodiaquine, Chloroquine, Quinine
Antifungal Agents	Amphotericin B, Flucytosine
Miscellaneous	Chlorpheniramine

ACE: angiotensin converting enzyme inhibitors.

**Table 2:** Types of drugs involved and their clinical manifestations in patients aged ≥75 years, followed-up for idiosyncratic drug-induced agranulocytosis cohort study of the University Hospital of Strasbourg (1984–2014) [54] *n* = 61.

Types of Drugs	*n* (%)	Clinical Manifestations	*n* (%)
Antiplatelet Agents	16 (23%)	Isolate Fever	17 (27.6%)
Antibacterials	15 (22%)	Septicemia or Septic Shock	15 (24.1%)
Antithyroid Drugs	12 (17%)	Pneumonia	12 (20.7%)
Antipsychotics/Antiepileptic Drugs	10 (14%)	Other Deep Infections	15 (24.1%)
NSAIDS	8 (12%)	Acute Tonsillitis	4 (6%)
Others	7 (11%)	Death	9 (14.8%)
		Asymptomatic	2(3.6%)

NSAIDS, nonsteroidal anti-inflammatory drugs.
